# Editorial: Coronavirus Disease (COVID-19): The Impact on Psychology of Sustainability, Sustainable Development, and Global Economy

**DOI:** 10.3389/fpsyg.2022.811863

**Published:** 2022-02-04

**Authors:** Muddassar Sarfraz, Ilhan Ozturk, Syed Ghulam Meran Shah

**Affiliations:** ^1^College of International Students, Wuxi University, Wuxi, China; ^2^Faculty of Business and Economics, Cag University, Mersin, Turkey; ^3^Faculty of Social Sciences, University of Castilla La Mancha, Cuenca, Spain

**Keywords:** COVID-19, sustainable development, global economy, financial markets psychology, quality of life keyword

## Introduction

The novel coronavirus disease (COVID-19) has rapidly spread across the entire globe, thus threatening human civilization. In 2019, the infectious disease was initiated from Wuhan, the city of China, leading the flare-up of the COVID-19 widespread to wagered the world socio-economic foundation. The terrifying characteristics of this pernicious disease have left an erratic impact on the world's safety conditions. In recent years, the increased epidemic has imposed magnificent challenges to worldwide societies, compelling them to experience unprecedented consequences in terms of transitory disclosures, travel confinements, and social distancing. The high COVID-19 susceptibility has imposed health challenges for the general population. The increasing COVID-19 fear has made the entire world bear psychological challenges, affecting the world's populace.

Indeed, the vindictive repercussions of the pandemic outbreak have intensified the damage while causing severe turbulence in the global economic sector. The expanding pandemic (COVID-19) has halted human settlement, causing the entire world to experience adverse outcomes of the epidemic. The unwanted ramification of the pandemic has compelled societies to battle against its intensity, affecting the physical and psychological aspects of individuals' well-being. Undoubtedly, the pandemic (COVID-19) outbreak has caused immense global disruptions' (e.g., social, economic, and health) in various sectors, thus affecting sustainable development.

Psychological sustainability development determines the standards of living where the quality of life influences the individual's health (i.e., physical and psychological). Arguably, the COVID-19 widespread has created global chaos, thereby damaging the world's economic foundation. The increasing uncertainty of pandemic has drastically altered the individuals' way of living (i.e., quality of life), severely affecting the socio-economic sustainability of global economies. Indeed, the abrupt changes initiated by the pandemic have affected individuals' quality of life while psychologically influencing sustainability development.

Due to the expanding significance of this Research Topic, the call for a special issue had submitted. The special issue aimed to determine the effect of COVID-19 on individuals' psychological wellbeing and economic sustainability. Further, it examined the socio-economic status affecting the individual lifestyle, especially the vulnerable groups (e.g., health workers, students, and professionals). Hence, the psychology of sustainable development states that the COVID-19 pandemic has drastically influenced global economies.

Moved by the COVID-19 uncertainty, the editors proposed the following topic *Coronavirus Disease (COVID-19): The Impact on Psychology of Sustainability, Sustainable Development, and Global Economy* in which new thoughts and insight had stimulated and incorporated in the COVID-19 context. Perhaps, in response, several pioneers' studies heeded the call, thus supporting the intended development with their valuable contributions. All the papers were successfully published, enriching the literature and accelerating future possibilities toward the topic development.

This Research Topic brings together 24 relevant articles fundamentally fulfilling the research aims. The papers written by the authors belong to different disciplinary sectors. The contributions from diverse geographical regions extend the scope of the research by empirically questioning the impact of COVID-19 from the social and economic perspective. Specifically, the articles probe the research from various geographical places, such as Vietnam, the United States, Europe, China, Turkey, Italy, and Germany. Indeed, the research articles offer a different perspective of the impact of COVID-19 on the psychology of sustainability through promoting interdisciplinary research in the field of Social Sciences and Occupational Health and Safety.

Furthermore, the additions made to the Research Topic present a comprehensive view of research design, excavating a significant understanding of the psychology of sustainable development during the COVID-19 period. The extensive range of articles provides new avenues for future researchers. In particular, these distinct themes have embedded psychological and sustainable context in the COVID-19 world. Thus, this editorial article presents a brief overview of all the papers organized under the Research Topic.

Unsurprisingly, the COVID-19 pandemic has severely damaged the worldwide economic foundation (Schneider et al., 2021). Shehzad, et al. state that COVID-19 effects on socio-economic activities have recorded a decline in the world's GDP, capital flows, and investment opportunities. The research showed that financial instability is not limited to economic loss only. But it also influences the psychology of sustainable development. In explanation, the authors stated that the COVID-19 picture lacked socio-economic development, directly affecting the individual's psychological sustainability. Indeed, the study concluded that COVID-19 had shaken the global marketplace by making the nations report a global market meltdown.

In addition, the other study shows that the devastating impact of coronavirus has ruined the individual's psychological health causing the vulnerable population to suffer its intended consequences in the shape of poor living (Xiang et al., 2020). However, by considering the perspective of various authors, the research illustrates that COVID-19 repercussions have significantly compelled the global economy to bear the negative consequences of the pandemic on household income, employment, and health status, subsequently influencing the quality of life. The study featured by Tran et al. shows COVID-19 has made the global economies experience huge income loss, thus adversely affecting the socio-economic status and individuals' wellbeing.

Furthermore, in line with the above research, the study demonstrated that the COVID-19 pandemic has drastically halted the global industries (Abbas et al., 2021), making the hospitality and tourism industry experience a declining effect. The staggering impact of the pandemic has taken a severe toll on individual psychological and physical health (Alonzi et al., 2020), thus causing them to face unprecedented consequences in the shape of economic stress and social isolation. According to Teng et al. the psychological impact of the pandemic (COVID-19) susceptibility has transformed the employees' lives by risking their mental wellbeing. Likewise, concerning the effect of sustainable psychological health, the study by Keshky et al. discusses the importance of improved psychological performance. Perhaps, both the studies report that the COVID-19 vulnerability has adversely affected individuals' mindset, unfavorably influencing individuals' psychological stability. Moreover, the fifth article indicates that financial resources are the most significant factors accelerating the nations' socio-economic progress. In recent years, the coronavirus outbreak has increased financial instability (Wilmarth, 2021), affecting the world's economic sustainable development. The study conducted by Castiglioni and Lozza shows the financial volatility has promoted prosocial choices such as compliance with the tax payment and giving charity to the general public, thus influencing economic stability.

In contrast, the COVID-19 pandemic has dramatically affected human lives, eventually influencing psychological sustainability development. This ultimate effect has deteriorated the living standards, thereby threatening the individual's psychological health. In explaining this notion, Çelmeçe and Menekay conducted a study on healthcare professionals and found that prolonged working hours have raised anxiety among individuals. Besides this, during the COVID-19 pandemic, the development projects (e.g., CPEC) have initiated colossal socio-economic benefits for nations like Pakistan and China. The research presented by Li, et al. reveals that the pandemic made the business enterprises perform exceptionally well by considerably contributing to the country's (i.e., Pakistan) economic sustainability. Surprisingly, this article provides a novel insight regarding the positive effect of the pandemic, subsequently fostering the country's sustainable development.

Additionally, the research shows that during the COVID-19 widespread, the high pace of work intensifies the effect of stress, anxiety, and burnout in the health workers, ultimately damaging their quality of living (Vafaei et al., 2020). By reviewing the home-quarantine activities of students', the researchers state that psychological distresses have disturbed students' social lives. Li, et al. showed that the feeling of helplessness, loneliness, and anxiety had changed the lifestyles while positively manifesting the symptoms of depression among the individuals. Indeed, the information gathered from this article records that increased health issues have a constant effect on individual psychological health, subsequently affecting the psychological sustainability development (i.e., quality of life).

However, in the wake of the pandemic, the COVID-19 restrictions (e.g., traveling, social) posed substantial challenges to achieving sustainable development goals. The ninth article holds paramount importance in highlighting the damaging effects of the widespread, thereby suggesting novel ways of mitigating the COVID-19 socio-economic adversity. Richter, Gabe-Thomas, et al. state that the COVID-19 pandemic has increased the strain on individuals' health, climate change, and socio-economic activities. Hence, the authors encourage promoting new practices, minimizing the COVID-19 effect, thus gaining long-term sustainable development. Moreover, Naseem et al. also support the findings by stating that the global outbreak has damaged the socio-economic conditions, paving the way toward the financial meltdown. The economic and health crises related to COVID-19 widespread had derived the behavior of the financial markets creating unbearable psychological resilience among the individuals' (i.e., health professionals). Verily, both the articles provided crucial information for future researches.

Considerably, several researchers have studied the prolonged effect of the global pandemic on various topics such as demographic characteristics, psychological effects, and mortality rates (Bonanad et al., 2020). In the illustration, the study conducted by Bilgili et al. revealed that the older population between the age of 65–70 were significantly affected by the deadly spread of the pandemic. Furthermore, the authors also suggested that government should adopt strict policies to control the increasing rate of worldwide deaths. In addition to this, COVID-19 has strengthened the work-life conflict by increasing the workload on the labor force. This statement has supported by Abdullah et al. who advise introducing social and psychological rewards to boost the employees' loyalty and job performance. Hence, these recommendations tremendously add value for achieving the sustainable goal during the pandemic.

Undoubtedly, the Coronavirus adverse impact has opened the black box of psychological sustainability, slowing down the market trend. The study by Ain et al. states that the pandemic has led the countries to experience an economic slowdown. In particular, the economic and health crises related to COVID-19 widespread had derived the behavior of the financial markets creating unbearable psychological resilience among the individuals' (i.e., health professionals). In explanation, other authors' Shehzad, et al. state that COVID-19 negativity has urged the investors to experience anxiety due to the drastic value decrease in the stock market. Indeed, the COVID-19 global pandemic has overburdened the world's economic system. Given the explanation, Szulc and Smith suggest that government must subsidize the employees' wages to reduce the workload, thus enhancing the employees' abilities and motivations to work. Perhaps, in the same vein, Dragan et al. illustrate that investing in the financial and psychological factors is essential in achieving organizational innovation. The study states that motivation, learning attitude, and behavior play a significant role in recording firms' sustainable development. In support, Güler and Haseki, in their study, also explained that positive psychological factors during the lockdown days have resulted in workers' motivation and happiness, thus enhancing individual's culinary skills and knowledge, ultimately contributing toward self-development. Potentially, all these articles emphasize achieving sustainability development by understanding the profound effects of the pandemic.

In contrast, the study by Hou et al. states that the increasing market volatility has significantly influenced the prices of Bitcoin currency. As a result, these progressing market inefficiencies have imposed immense psychological pressure on the investors, thus affecting market sustainability. Furthermore, in response to the COVID-19 vulnerability, the protection measures implemented by the government has limited businesses activities. Consistent with the given statement, the research by Xiang et al. showed that economic losses during the pandemic have resulted in global economies looking up to the future recession. The catastrophic consequences of the pandemic (i.e., poverty and unemployment) have badly affected business activities, driving the need for economic rehabilitation. In support, Richter, Richter, et al. call for quick action (e.g., government fund policies, building knowledge, and developing tailor-made solutions) for strengthening future opportunities.

The COVID-19 pandemic had a hard hit on the world's economy, leading numerous industries to face its vulnerability in the shape of declining revenues and earnings (Ozili and Arun, 2020). The economic environment of the pandemic has raised multiple questions on the expanding financial anxieties. This escalating market volatility during the COVID-19 has stopped the growth and development in many countries. Basyouni and Keshky explains that COVID-19 has provoked financial distress among the employees. In particular, this increased stress and depression have directly affected individuals' quality of life. The paper by Obrenovic et al. explained that the underlying mechanism is needed to control the COVID-19 events, heightening the psychological challenges.

However, among the COVID-19 vulnerability, financial instability and future unpredictability are the most significant factors affecting the psychological conditions, thus weakening economic sustainability. The growing volatility has worsened the global economies', thus fading the worldwide economic structure. Consistently, many studies indicated job insecurity to be the prime factor contributing to financial instability. The pandemic has made most people unemployed (Petrosky-Nadeau and Valletta, 2020). The study conducted by Wojtkowska et al. illustrates that the dread of COVID-19 vulnerabilities had caused severe disruptions in individuals' lives. Perhaps, the above studies help individuals understand the COVID-19 emerging factors (e.g., psychological and financial) linked to an individual's wellbeing and the country's economic progress.

In addition to this, the last paper reveals that the pandemic has also provided numerous benefits to firms' by accelerating their innovation process (Gopalakrishnan and Kovoor-Misra, 2021). Jialu et al. concluded that enterprise participation had enhanced the firm's technological innovation performance by colossally bringing advantages based on the firm's knowledge characteristics. This inevitable knowledge trend has remarkably increased the structural holes, thus strengthening the enterprise position in organization sustainable performance.

Undoubtedly, the COVID-19 pandemic has caused severe disruption to the worldwide economies', thereby compelling individuals and firms to face its psychological and financial vulnerabilities. Indeed, all the articles highlight the need to adopt transformational development solutions for restoring individuals' psychological wellbeing and the world's economic sustainability.

We hope that all 24 papers selected under the intended topic expand the understanding of the effect of COVID-19 psychological sustainability on the socio-economic context. All the published papers add value for the general public, professionals, and future researchers, thus inspiring them to improve their lives while valuably adding to the desired research. All these contributions will serve as a guide model for future researchers, making them challenge this complex phenomenon of COVID-19 psychology.

## Author Contributions

All authors listed have made a substantial, direct, and intellectual contribution to the work and approved it for publication.

## Conflict of Interest

The authors declare that the research was conducted in the absence of any commercial or financial relationships that could be construed as a potential conflict of interest.

## Publisher's Note

All claims expressed in this article are solely those of the authors and do not necessarily represent those of their affiliated organizations, or those of the publisher, the editors and the reviewers. Any product that may be evaluated in this article, or claim that may be made by its manufacturer, is not guaranteed or endorsed by the publisher.
